# Naphthalene-2,3-diol–imidazole (1/1)

**DOI:** 10.1107/S1600536808025555

**Published:** 2008-08-13

**Authors:** Yong-Tao Wang, Gui-Mei Tang, Wen-Zhu Wan

**Affiliations:** a Department of Chemical Engineering, Shandong Institute of Light Industry, Jinan, Shandong 250353, People’s Republic of China

## Abstract

In the title cocrystal, C_10_H_8_O_2_·C_3_H_4_N_2_, inter­molecular O—H⋯O and N—H⋯O hydrogen bonds connect the naphthalene-2,3-diol and imidazole mol­ecules into a two-dimensional supra­molecular framework.

## Related literature

For other cocrystals of naphthalene-2,3-diol, see: Fritchie & Johnston (1975 ▶); Wang & Tang (2006 ▶); Wang, Tang & Ng (2006 ▶); Wang, Tang & Wan (2006 ▶); Wells *et al.* (1974 ▶).
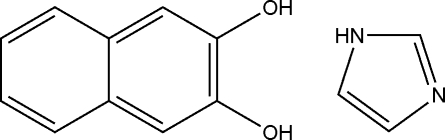


## Experimental

### 

#### Crystal data

C_10_H_8_O_2_·C_3_H_4_N_2_
*M* *_r_* = 228.25
Orthorhombic, 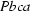

*a* = 12.0003 (17) Å
*b* = 7.7862 (11) Å
*c* = 25.863 (4) Å
*V* = 2416.6 (6) Å^3^
*Z* = 8
Mo *K*α radiation
μ = 0.09 mm^−1^
*T* = 296 (2) K
0.30 × 0.30 × 0.20 mm

#### Data collection

Bruker SMART diffractometer
Absorption correction: none
18637 measured reflections
2777 independent reflections
2142 reflections with *I* > 2σ(*I*)
*R*_int_ = 0.031

#### Refinement

*R*[*F* ^2^ > 2σ(*F* ^2^)] = 0.041
*wR*(*F* ^2^) = 0.125
*S* = 1.04
2777 reflections
166 parameters
3 restraints
H atoms treated by a mixture of independent and constrained refinement
Δρ_max_ = 0.16 e Å^−3^
Δρ_min_ = −0.20 e Å^−3^

### 

Data collection: *SMART*; cell refinement: *SAINT* (Bruker, 2001 ▶); data reduction: *SAINT*; program(s) used to solve structure: *SHELXS97* (Sheldrick, 2008 ▶); program(s) used to refine structure: *SHELXL97* (Sheldrick, 2008 ▶); molecular graphics: *SHELXTL* (Sheldrick, 2008 ▶); software used to prepare material for publication: *SHELXTL*.

## Supplementary Material

Crystal structure: contains datablocks global, I. DOI: 10.1107/S1600536808025555/ng2483sup1.cif

Structure factors: contains datablocks I. DOI: 10.1107/S1600536808025555/ng2483Isup2.hkl

Additional supplementary materials:  crystallographic information; 3D view; checkCIF report

## Figures and Tables

**Table 1:** Hydrogen-bond geometry (Å, °)

*D*—H⋯*A*	*D*—H	H⋯*A*	*D*⋯*A*	*D*—H⋯*A*
O1—H1*B*⋯O2^i^	0.92 (2)	1.78 (2)	2.6877 (14)	166 (2)
O2—H2*A*⋯N1	1.03 (2)	1.57 (2)	2.5947 (15)	170 (2)
N2—H2*B*⋯O1^ii^	0.89 (2)	2.09 (3)	2.9185 (19)	156 (2)

Symmetry codes: (i) 
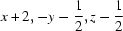
; (ii) 
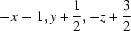
.

**Fig. 1.**
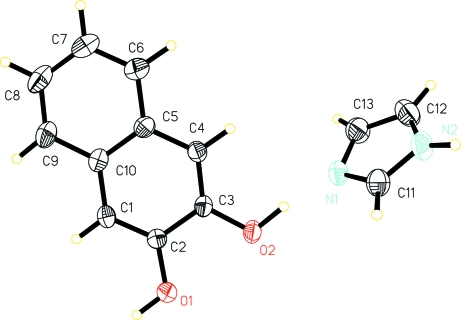
A drawing of (I), with the atom numbering scheme. Displacement ellipsoids are drawn at the 30% probability level.

**Fig. 2.**
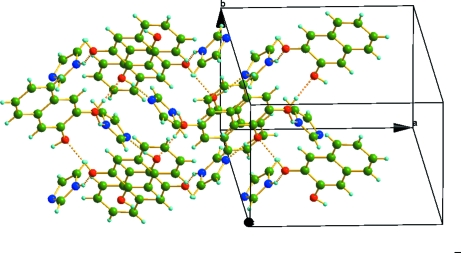
Packing diagram of (I); hydrogen bonds are shown by dashed lines.

## supplementary crystallographic information

### Comment

During past decade, the field of molecular co-crystals have received considerable attention, for example, the design, construction, properties and the definition of molecular co-crystals, partly because co-crystallization reactions offer unique opportunities for examining the balance between and structural influence of intermolecular interactions. Recently, a lot of co-crystals containing some organic acids and bases, have been successfully synthesized and characterized by our research group. Especially, co-crystals containing naphthalene-2,7-diol with some organic bases have been prepared and reported (Wang & Tang, 2006; Wang, Tang & Ng, 2006; Wang, Tang & Wan, 2006). A series of supramolecular structures of self-assembly with different motifs have been obtained. There are a few co-crystals about naphthalene-2,3-diol (ndo) as organic acid; some interesting structures have been generated through supramolecular self-assemblies (Fritchie & Johnston, 1975; Wells, *et al.*, 1974). To study a series of co-crystals containing ndo and to further explore its properties, we have selected the structure of the co-crystal, (I), of ndo and imidazole.

A view of the title structure is shown in Fig. 1. The asymmetric unit consists of one independent ndo molecule and one independent molecule of imidazole. In the crystal structure of the title compound, intermolecular O—H···O and N—H···O hydrogen bonds connect naphthalene-2,3-diol molecules and imidazole molecules into a linear ribbon motif, which are further extended to two-dimensional supramolecular framwork through N—H···O hydrogen bonds (Table 1, Fig. 2).

### Experimental

A mixture of naphthalene-2,3-diol (80 mg, 0.5 mmol) and imidazole (34 mg, 0.5 mmol) was recrystallized from methanol (5 ml) and water (1 ml) (yield: 102 mg, 90%), from which a yellow needle suitable for *x*-ray diffraction was selected. Analysis found (%): C 68.21; H, 5.33; N, 12.21; requires (%): C, 68.41; H, 5.30; N, 12.27.

### Refinement

All H atoms were located in a difference Fourier map. Carbon-bound hydrogen atoms were positioned geometrically (C—H = 0.93 A °), and were included in the refinement in the riding-model approximation, with *U*_iso_(H) = 1.2Ueq(C). Oxygen- and nitrogen-bound hydrogen atoms were restrained and refined independently, with isotropic displacement parameters, giving final O—H and N—H distances in the range 0.895 (5)–0.911 (9), 0.897 (10) A °, respectively.

### Crystal data

**Table:**

C_10_H_8_O_2_·C_3_H_4_N_2_	*F*_000_ = 960
*M**_r_* = 228.25	*D*_x_ = 1.255 Mg m^−^^3^
Orthorhombic, *P**b**c**a*	Mo *K*α radiation λ = 0.71073 Å
Hall symbol: -P 2ac 2ab	Cell parameters from 5055 reflections
*a* = 12.0003 (17) Å	θ = 2.3–26.3º
*b* = 7.7862 (11) Å	µ = 0.09 mm^−^^1^
*c* = 25.863 (4) Å	*T* = 296 (2) K
*V* = 2416.6 (6) Å^3^	Column, yellow
*Z* = 8	0.30 × 0.30 × 0.20 mm

### Data collection

**Table:**

Bruker SMART diffractometer	2142 reflections with *I* > 2σ(*I*)
Radiation source: fine-focus sealed tube	*R*_int_ = 0.031
Monochromator: graphite	θ_max_ = 27.6º
*T* = 296(2) K	θ_min_ = 1.6º
φ and ω scans	*h* = −13→15
Absorption correction: None	*k* = −10→9
18637 measured reflections	*l* = −33→33
2777 independent reflections	

### Refinement

**Table:**

Refinement on *F*^2^	Secondary atom site location: difference Fourier map
Least-squares matrix: full	Hydrogen site location: inferred from neighbouring sites
*R*[*F*^2^ > 2σ(*F*^2^)] = 0.041	H atoms treated by a mixture of independent and constrained refinement
*wR*(*F*^2^) = 0.125	*w* = 1/[σ^2^(*F*_o_^2^) + (0.0675*P*)^2^ + 0.2922*P*] where *P* = (*F*_o_^2^ + 2*F*_c_^2^)/3
*S* = 1.04	(Δ/σ)_max_ < 0.001
2777 reflections	Δρ_max_ = 0.16 e Å^−^^3^
166 parameters	Δρ_min_ = −0.20 e Å^−^^3^
3 restraints	Extinction correction: none
Primary atom site location: structure-invariant direct methods	

### Special details

**Table:**

Geometry. All e.s.d.'s (except the e.s.d. in the dihedral angle between two l.s. planes) are estimated using the full covariance matrix. The cell e.s.d.'s are taken into account individually in the estimation of e.s.d.'s in distances, angles and torsion angles; correlations between e.s.d.'s in cell parameters are only used when they are defined by crystal symmetry. An approximate (isotropic) treatment of cell e.s.d.'s is used for estimating e.s.d.'s involving l.s. planes.
Refinement. Refinement of *F*^2^ against ALL reflections. The weighted *R*-factor *wR* and goodness of fit *S* are based on *F*^2^, conventional *R*-factors *R* are based on *F*, with *F* set to zero for negative *F*^2^. The threshold expression of *F*^2^ > σ(*F*^2^) is used only for calculating *R*-factors(gt) *etc*. and is not relevant to the choice of reflections for refinement. *R*-factors based on *F*^2^ are statistically about twice as large as those based on *F*, and *R*- factors based on ALL data will be even larger.

### Fractional atomic coordinates and isotropic or equivalent isotropic displacement parameters (Å^2^)

**Table:**

	*x*	*y*	*z*	*U*_iso_*/*U*_eq_	
O1	0.75477 (8)	0.63170 (13)	0.59205 (4)	0.0517 (3)	
H1B	0.8003 (12)	0.7228 (17)	0.5918 (7)	0.074 (5)*	
O2	0.60386 (7)	0.39458 (12)	0.57667 (4)	0.0519 (3)	
H2A	0.5427 (12)	0.333 (2)	0.5673 (8)	0.094 (6)*	
C1	0.63552 (11)	0.78573 (16)	0.65109 (5)	0.0438 (3)	
H1A	0.6909	0.8665	0.6575	0.053*	
C2	0.65645 (10)	0.65273 (15)	0.61825 (4)	0.0396 (3)	
C3	0.57423 (10)	0.52524 (15)	0.60887 (4)	0.0404 (3)	
C4	0.47199 (11)	0.54089 (16)	0.63144 (5)	0.0455 (3)	
H4	0.4176	0.4587	0.6247	0.055*	
C5	0.44700 (11)	0.67966 (17)	0.66483 (5)	0.0443 (3)	
C6	0.34112 (12)	0.6975 (2)	0.68858 (6)	0.0570 (4)	
H6	0.2849	0.6194	0.6809	0.068*	
C7	0.32087 (14)	0.8271 (2)	0.72241 (6)	0.0671 (5)	
H7	0.2514	0.8359	0.7381	0.081*	
C8	0.40329 (16)	0.9469 (2)	0.73374 (6)	0.0705 (5)	
H8	0.3886	1.0345	0.7572	0.085*	
C9	0.50538 (14)	0.9372 (2)	0.71078 (5)	0.0594 (4)	
H9	0.5592	1.0195	0.7183	0.071*	
C10	0.53038 (11)	0.80258 (16)	0.67555 (4)	0.0435 (3)	
C11	0.40496 (13)	0.1697 (2)	0.50288 (5)	0.0577 (4)	
H11	0.4295	0.2319	0.4744	0.069*	
C12	0.31524 (16)	−0.0138 (3)	0.54918 (7)	0.0804 (5)	
H12	0.2671	−0.1007	0.5597	0.096*	
C13	0.38620 (14)	0.0743 (2)	0.57906 (6)	0.0673 (4)	
H13	0.3955	0.0583	0.6144	0.081*	
N1	0.44264 (9)	0.19072 (14)	0.54994 (4)	0.0504 (3)	
N2	0.32741 (12)	0.0486 (2)	0.50092 (5)	0.0682 (4)	
H2B	0.2928 (17)	0.018 (3)	0.4716 (6)	0.113 (8)*	

### Atomic displacement parameters (Å^2^)

**Table:**

	*U*^11^	*U*^22^	*U*^33^	*U*^12^	*U*^13^	*U*^23^
O1	0.0449 (5)	0.0484 (6)	0.0619 (6)	−0.0061 (4)	0.0087 (4)	−0.0098 (4)
O2	0.0416 (5)	0.0462 (5)	0.0678 (6)	0.0014 (4)	−0.0028 (4)	−0.0219 (4)
C1	0.0484 (7)	0.0386 (7)	0.0445 (6)	−0.0042 (5)	−0.0078 (5)	−0.0028 (5)
C2	0.0384 (6)	0.0395 (7)	0.0409 (6)	0.0014 (5)	−0.0018 (5)	0.0006 (5)
C3	0.0417 (7)	0.0356 (6)	0.0439 (6)	0.0030 (5)	−0.0058 (5)	−0.0050 (5)
C4	0.0405 (7)	0.0401 (7)	0.0561 (7)	−0.0026 (5)	−0.0010 (5)	−0.0056 (6)
C5	0.0454 (7)	0.0442 (7)	0.0432 (6)	0.0066 (6)	−0.0012 (5)	0.0003 (5)
C6	0.0497 (8)	0.0621 (9)	0.0593 (8)	0.0064 (7)	0.0059 (6)	−0.0005 (7)
C7	0.0620 (10)	0.0788 (11)	0.0605 (9)	0.0202 (9)	0.0108 (7)	−0.0058 (8)
C8	0.0773 (12)	0.0754 (11)	0.0588 (9)	0.0247 (9)	−0.0010 (8)	−0.0242 (8)
C9	0.0659 (10)	0.0562 (9)	0.0560 (8)	0.0088 (7)	−0.0103 (7)	−0.0176 (7)
C10	0.0505 (7)	0.0406 (7)	0.0394 (6)	0.0072 (5)	−0.0069 (5)	−0.0025 (5)
C11	0.0628 (9)	0.0592 (9)	0.0511 (7)	0.0000 (7)	−0.0112 (7)	−0.0014 (6)
C12	0.0776 (12)	0.0805 (12)	0.0830 (12)	−0.0316 (10)	0.0031 (9)	−0.0055 (10)
C13	0.0733 (11)	0.0780 (11)	0.0506 (8)	−0.0107 (9)	−0.0028 (7)	−0.0013 (7)
N1	0.0504 (6)	0.0479 (6)	0.0528 (6)	0.0003 (5)	−0.0107 (5)	−0.0094 (5)
N2	0.0618 (8)	0.0771 (10)	0.0656 (8)	−0.0072 (7)	−0.0184 (7)	−0.0204 (7)

### Geometric parameters (Å, °)

**Table:**

O1—C2	1.3705 (15)	C7—C8	1.390 (3)
O1—H1B	0.895 (9)	C7—H7	0.9300
O2—C3	1.3620 (14)	C8—C9	1.363 (2)
O2—H2A	0.911 (9)	C8—H8	0.9300
C1—C2	1.3627 (17)	C9—C10	1.4207 (18)
C1—C10	1.4176 (18)	C9—H9	0.9300
C1—H1A	0.9300	C11—N1	1.3089 (17)
C2—C3	1.4204 (17)	C11—N2	1.326 (2)
C3—C4	1.3642 (17)	C11—H11	0.9300
C4—C5	1.4154 (18)	C12—C13	1.339 (2)
C4—H4	0.9300	C12—N2	1.347 (2)
C5—C10	1.4120 (19)	C12—H12	0.9300
C5—C6	1.4180 (18)	C13—N1	1.359 (2)
C6—C7	1.358 (2)	C13—H13	0.9300
C6—H6	0.9300	N2—H2B	0.897 (10)
			
C2—O1—H1B	115.7 (11)	C9—C8—C7	120.68 (14)
C3—O2—H2A	110.3 (13)	C9—C8—H8	119.7
C2—C1—C10	120.83 (11)	C7—C8—H8	119.7
C2—C1—H1A	119.6	C8—C9—C10	120.65 (15)
C10—C1—H1A	119.6	C8—C9—H9	119.7
C1—C2—O1	123.90 (11)	C10—C9—H9	119.7
C1—C2—C3	120.63 (11)	C5—C10—C1	118.71 (11)
O1—C2—C3	115.47 (10)	C5—C10—C9	118.44 (13)
O2—C3—C4	124.27 (11)	C1—C10—C9	122.85 (13)
O2—C3—C2	116.43 (11)	N1—C11—N2	111.53 (14)
C4—C3—C2	119.29 (11)	N1—C11—H11	124.2
C3—C4—C5	121.32 (12)	N2—C11—H11	124.2
C3—C4—H4	119.3	C13—C12—N2	106.33 (15)
C5—C4—H4	119.3	C13—C12—H12	126.8
C10—C5—C4	119.15 (12)	N2—C12—H12	126.8
C10—C5—C6	118.92 (12)	C12—C13—N1	109.81 (15)
C4—C5—C6	121.92 (13)	C12—C13—H13	125.1
C7—C6—C5	120.81 (15)	N1—C13—H13	125.1
C7—C6—H6	119.6	C11—N1—C13	105.05 (12)
C5—C6—H6	119.6	C11—N2—C12	107.28 (12)
C6—C7—C8	120.46 (15)	C11—N2—H2B	123.1 (15)
C6—C7—H7	119.8	C12—N2—H2B	129.6 (15)
C8—C7—H7	119.8		
			
C10—C1—C2—O1	177.81 (11)	C7—C8—C9—C10	−1.2 (2)
C10—C1—C2—C3	−1.71 (18)	C4—C5—C10—C1	2.02 (18)
C1—C2—C3—O2	−178.13 (11)	C6—C5—C10—C1	−179.01 (11)
O1—C2—C3—O2	2.31 (16)	C4—C5—C10—C9	−177.49 (12)
C1—C2—C3—C4	2.72 (18)	C6—C5—C10—C9	1.48 (18)
O1—C2—C3—C4	−176.84 (11)	C2—C1—C10—C5	−0.67 (18)
O2—C3—C4—C5	179.59 (12)	C2—C1—C10—C9	178.82 (12)
C2—C3—C4—C5	−1.33 (19)	C8—C9—C10—C5	0.2 (2)
C3—C4—C5—C10	−1.02 (19)	C8—C9—C10—C1	−179.33 (14)
C3—C4—C5—C6	−179.96 (13)	N2—C12—C13—N1	−0.1 (2)
C10—C5—C6—C7	−2.1 (2)	N2—C11—N1—C13	0.76 (18)
C4—C5—C6—C7	176.80 (14)	C12—C13—N1—C11	−0.39 (19)
C5—C6—C7—C8	1.1 (2)	N1—C11—N2—C12	−0.8 (2)
C6—C7—C8—C9	0.6 (3)	C13—C12—N2—C11	0.6 (2)

### Hydrogen-bond geometry (Å, °)

**Table:**

*D*—H···*A*	*D*—H	H···*A*	*D*···*A*	*D*—H···*A*
O1—H1B···O2^i^	0.92 (2)	1.78 (2)	2.6877 (14)	166 (2)
O2—H2A···N1	1.03 (2)	1.57 (2)	2.5947 (15)	170 (2)
N2—H2B···O1^ii^	0.89 (2)	2.09 (3)	2.9185 (19)	156 (2)

Symmetry codes: (i) *x*+2, −*y*−1/2, *z*−1/2; (ii) −*x*−1, *y*+1/2, −*z*+3/2.
